# A descriptive study of samples sizes used in agreement studies published in the PubMed repository

**DOI:** 10.1186/s12874-022-01723-5

**Published:** 2022-09-19

**Authors:** Oscar Han, Hao Wei Tan, Steven Julious, Laura Sutton, Richard Jacques, Ellen Lee, Jen Lewis, Stephen Walters

**Affiliations:** School of Health and Related Research, University of Sheffield, Sheffield, UK

**Keywords:** Agreement, Design, Method comparison, Sample size, Test–retest

## Abstract

**Introduction:**

A sample size justification is required for all studies and should give the minimum number of subjects to be recruited for the study to achieve its primary objective. The aim of this review is to describe sample sizes from agreement studies with continuous or categorical endpoints and different methods of assessing agreement, and to determine whether sample size justification was provided.

**Methods:**

Data were gathered from the PubMed repository with a time interval of 28^th^ September 2018 to 28^th^ September 2020. The search returned 5257 studies of which 82 studies were eligible for final assessment after duplicates and ineligible studies were excluded.

**Results:**

We observed a wide range of sample sizes. Forty-six studies (56%) used a continuous outcome measure, 28 (34%) used categorical and eight (10%) used both. Median sample sizes were 50 (IQR 25 to 100) for continuous endpoints and 119 (IQR 50 to 271) for categorical endpoints. Bland–Altman limits of agreement (median sample size 65; IQR 35 to 124) were the most common method of statistical analysis for continuous variables and Kappa coefficients for categorical variables (median sample size 71; IQR 50 to 233). Of the 82 studies assessed, only 27 (33%) gave justification for their sample size.

**Conclusions:**

Despite the importance of a sample size justification, we found that two-thirds of agreement studies did not provide one. We recommend that all agreement studies provide rationale for their sample size even if they do not include a formal sample size calculation.

**Supplementary Information:**

The online version contains supplementary material available at 10.1186/s12874-022-01723-5.

## Background

Agreement is defined as the extent to which measurements or ratings are the same as one another. Inter-rater agreement is the similarity of measurements from different instruments or raters on the same subjects, and intra-rater agreement is the consistency of repeat measurements by the same instrument or rater on the same subjects [1]. Agreement studies in medical research include method comparison or test–retest studies to evaluate the techniques used in clinical evaluation. Their application includes fields of research such as medicine, surgery and radiology [2].

Agreement studies are important to facilitate the development of new clinical methods of evaluation, ensuring they are consistent with the current ‘gold standard’ approach, or to ensure diagnostic consistency between and within assessors. Agreement is commonly tested using statistical methods such as Bland–Altman limits of agreement (LoA), the intraclass correlation (ICC) and Kappa coefficients. However, methods inappropriate for the assessment of agreement are also often used [2].

Quantifying an appropriate sample size for research studies is important to prevent the recruited sample from being overly small or large. A small sample size can lead to inconclusive results with wide confidence limits, whereas a too large a sample could be expensive and time-consuming, study participants could be exposed to unnecessary burden, and it could be considered unethical as patients continue to be enrolled after a time when the research questions can be answered [3].

Determining the target sample size is an important step in any study design and should be considered and justified a priori. However, in the design of agreement studies, sample size determination often does not receive the same level of attention as the choice of method for assessing agreement [4, 5].

In this study we reviewed sample sizes used in agreement studies in the medical literature, and assessed whether the authors justified the sample size and conducted formal sample size estimation.

The research aims were:

1. To describe the sample sizes used or reported in clinical agreement studies with a categorical (binary or ordinal) or continuous endpoint;
2. To describe the sample sizes used in agreement studies when using different statistical methods to assess agreement;
3. To describe the use of formal sample size estimation and calculations in agreement studies.

## Methods

The PubMed repository (https://pubmed.ncbi.nlm.nih.gov, accessed 29^th^ September 2020) was used to identify medical research studies that investigated intra-rater or inter-rater agreement or method comparison between different clinical instruments using the same units of measurement. The time scope of the search result was two years between 28^th^ September 2018 and 28^th^ September 2020. An online search was conducted on 29^th^ September 2020 using the following search terms: ‘Agreement Study’ OR ‘Test Repeatability’ OR ‘Method Comparison’. Studies reporting agreement of categorical (binary or ordinal) or continuous variables were considered. The selection was limited to clinical studies relating to only human participants with full text available in the English language.

Search results were identified and exported to Microsoft Excel where duplicates were removed. We excluded studies that compared techniques that used different units of measurement and studies not involving human subjects. The selection of studies was conducted independently by two researchers (OH and HT). In the event of disagreement, a third researcher was to be called in for evaluation; however, no disagreement was found between the two researchers during the primary selection stage. The initial extraction of data for the analysis was conducted by the same two researchers.

After the initial extraction by OH and HT the data for each study was reviewed by two additional researchers (from EL, SJ, LS, SW, JL and RJ) and verified against the original source. If there was any disagreement on the final data extracted SJ and LS adjudicated with OH and HT. The data extracted from the papers were analysed by OH and HT.

Studies were categorised into four fields: medicine, surgery, radiology and allied health. Studies were also classified into five groups according to the main statistical method used to assess agreement:

1. Bland–Altman LoA
2. ICC
3. Kappa coefficients
4. Significance tests
5. Other methods (e.g. percent agreement, Pearson/Spearman correlation)

Further categorisation was made into types of endpoints: categorical and/or continuous.

Data pertaining to planned sample sizes, sample size estimation and actual sample sizes were identified. Where no planned sample size was given the actual sample size was reported. To describe the distribution of sample sizes, the mean, median, interquartile range and range were calculated.

We assessed whether sample size justification was provided. The justification could be through a formal sample size calculation or narratively to explain the rationale for the sample size.

## Results

**Fig. 1:**
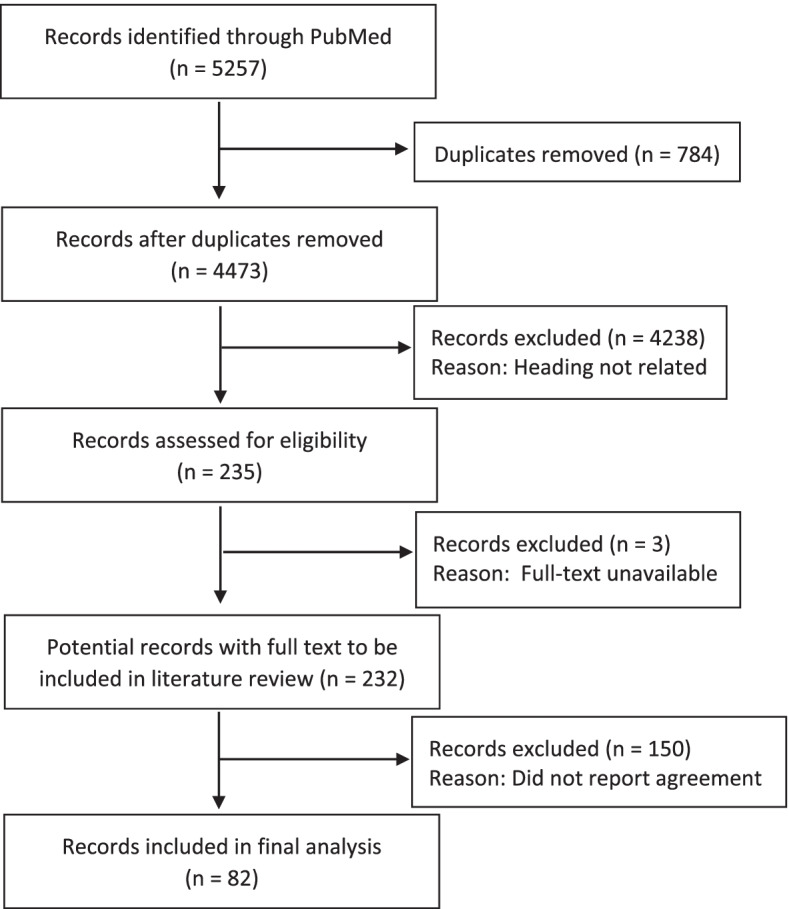
PRISMA Flow Diagram of Review Process

The PubMed repository search returned 5,257 studies. After removal of duplicates, 4,473 titles were screened. There were 235 titles eligible for further review based on heading relevancy. Three studies did not have full text available; their respective authors were contacted, however no reply was received and the studies were excluded. After exclusion of a further 150 ineligible studies that did not report agreement analyses, 82 studies were included in the present analysis. The study selection process is summarised in Fig. 1.

A summary of the characteristics of the 82 studies meeting the review inclusion criteria is presented in Table 1.

**Table 1:** Study characteristics of the 82 articles involved in final analysis

		Agreement Studies (*n* = 82)	
		n	%
Field of study	Allied Health	4	4.9
	Medicine	45	54.9
	Radiology	29	35.4
	Surgery	4	4.9
Sample size justification	Yes	27	32.9
	No	55	67.1
Endpoint	Categorical	28	34.1
	Continuous	46	56.1
	Both	8	9.8
Disease area	Cardiovascular	20	24.4
	Gastrointestinal	3	3.7
	Geriatrics	6	7.3
	Haematology	2	2.4
	Hepatology	5	6.1
	Mental Health	2	2.4
	Neurology	3	3.7
	Oncology	7	8.5
	Ophthalmology	6	7.3
	Orthopaedic	10	12.2
	Respiratory	2	2.4
	Urology	2	2.4
	Others	14	17.1

Each study reported the sample size used. However, only 27 out of 82 studies (33%) provided justification for the sample size for agreement analysis. Of the 27 studies that had a formal justification for the sample size, 22 (82%) showed evidence of sample size calculation having been performed, including parameter estimates and/or reference to formulae or software packages used. All but one of those 27 studies provided at least some parameter estimates, though not all provided sufficient information for precise replication. Of the five studies providing rationale but no formal calculation, sample sizes were determined by the study being nested within another powered on a different endpoint (*n* = 3), fixed by calendar time (data from a one-year period; *n* = 1), or selected based on the sample size of similar studies (*n* = 1).

**Fig. 2:**
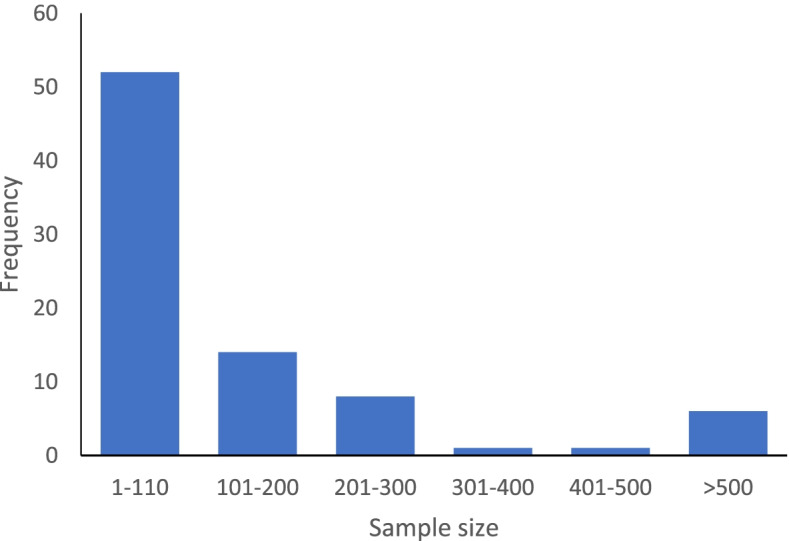
Histogram of sample sizes used in eligible studies for assessing agreement (*n* = 82)

A histogram showing the distribution of sample sizes across the 82 eligible studies is shown in Fig. 2. The median sample size was 62.5 (IQR: 35, 159; range: 10, 4469).

Sample sizes according to clinical research area, statistical methodology and type of endpoint are presented in Table 2. Further breakdowns of research area and methodology by type of endpoint are provided in Supplementary tables ST1 and ST2.

**Table 2:** Distribution of sample sizes according to field of study, statistical method and endpoint

			Sample size reported by studies			
		n	Median	Mean	Interquartile range	Range
Field of study	Allied Health	4	53.0	76.3	[45.0, 107.5]	[40, 159]
	Medicine	45	80.0	259.0	[39.5, 180.0]	[11, 4469]
	Radiology	29	50.0	206.2	[27.0, 142.5]	[10, 3082]
	Surgery	4	52.0	955.8	[18.5, 1893.0]	[13, 3706]
Sample size justification	Yes	27	50.0	70.6	[27.0, 75.0]	[12, 275]
	No	55	62.5	360.7	[35.3, 158.0]	[10, 4469]
Statistical method	Bland–Altman LoA	41	65.0	89.0	[34.5, 124.0]	[12, 278]
	ICC	29	42.0	221.7	[27.0, 64.5]	[12, 4469]
	Kappa coefficient	35	71.0	376.9	[50.0, 233.0]	[10, 3706]
	Significance test	20	54.5	86.2	[26.5, 128.0]	[12, 267]
	Other	32	57.0	430.7	[38.0, 124.5]	[10, 4469]
Endpoint	Continuous	46	49.5	74.8	[25.0, 100.0]	[11, 265]
	Categorical	28	119.0	448.1	[50.0, 271.0]	[10, 3706]
	Both	8	72.5	721.6	[59.0, 500.5]	[40, 4469]

*ICC* intraclass correlation, *LoA* limits of agreement

Studies classified under medicine tended to have larger sample sizes, with a median sample size of 80 (IQR 45 to 108). This was followed by allied health, surgery and lastly radiology with a median sample size of 50 (IQR 27 to 143).

Of the 82 research studies assessed, 30 studies (37%) utilised one statistical method to assess agreement whilst 52 studies (63%) utilised two or more statistical methods. Bland–Altman LoA was the most used statistical method by studies measuring continuous endpoints (41 studies; 50%) and Kappa coefficients were most used by studies measuring categorical endpoints (35 studies; 43%).

Studies in which agreement was assessed using the Kappa method had the largest median sample size of 71 (IQR 50 to 233) and those using the ICC as the primary method had the smallest median sample size of 42 (IQR 27 to 65). For significance tests, the most common approach was a paired *t*-test, used in seven studies. The most common ‘other’ statistical method employed was a correlation coefficient, used in seven studies.

Overall, studies measuring primarily categorical endpoints had a larger median sample size of 119 (IQR 50 to 271), compared to those focussing primarily on continuous endpoints, with a median of 50 (IQR 25 to 100). It was noted that all median sample sizes were smaller than mean sample sizes, indicative of positively skewed sample size distributions.

## Discussion

Our review of the PubMed repository identified 82 eligible agreement studies published in the medical literature between 2018 and 2020. The studies covered a variety of disease areas. We observed a wide range of sample sizes and variability in typical sample size according to clinical field, statistical method and type of endpoint.

Continuous endpoints were the more common, for which Bland–Altman LoA was the most frequent statistical approach used, with a median sample size of 89 (IQR 35 to 124). Finding Bland–Altman LoA the most common approach is consistent with the review of Zaki et al. [2]. Another finding consistent with their review is our observation of the continued use of the correlation coefficient, despite it being deemed inappropriate for the assessment of agreement [6]. However, we did observe a lower frequency of use.

We found Kappa statistics to be the most common approach used with categorical variables, with a median sample size of 71 (IQR 50 to 233). Kappa is commonly used for the assessment of agreement using binary and ordinal scales [7]. Studies with categorical variables tended to have larger sample sizes than those focussing mainly on continuous variables. The finding of larger sample sizes for categorical compared to continuous outcomes is consistent with research in the context of pilot studies [8] and definitive outcome trials, as inferred from the target standardised effect sizes reported by Rothwell et al. [9].

We found that all included studies reported a sample size, but only one-third provided justification for their sample size, and of those, not all reported use of statistical sample size formulae. Kottner et al. [1] recommended that sample size justification be made explicit in agreement studies to ensure transparency and credibility. Despite this, Farzin et al. [10] found justification for the sample size was given in only nine of 280 agreement studies (3%) conducted in diagnostic imaging journals, which is markedly lower than we observed in the present review.

Variation in the quality of sample size reporting has been examined in the context of clinical trials, with 95% of the trails published in high impact journals reviewed by Charles et al. [11] reporting sample size calculations, but only 53% reporting all parameters required for replication. Copsey et al. [12] reported a lower proportion of trials describing a sample size calculation at 67%, with only 21% reporting all the components of the calculation. Tulka et al. [13] reported that just 42% of trials justified their sample size, and only 21% described a complete sample size calculation. Sample size reporting in clinical trials could be expected to be of higher quality since publication of the first CONSORT guidance in 1996 [14]. The trial reviews show higher proportions of studies reporting details of sample size estimation compared to agreement studies, but that inadequate reporting remains prevalent. The higher proportion of studies providing sample size details reported by Charles et al. [11] was likely because their review included only the highest impact medical journals.

Some authors suggest general rules of thumb for sample sizes for agreement studies, for example, Liao [4] recommended a minimum sample size of 32 and McAlinden et al. [15] a minimum sample size of 100 for agreement studies measuring continuous variables. A preferred approach, where possible, would be to use specific calculations that take into account the research question and appropriate statistical method of analysis. Formulae to determine minimum sample size requirements are available for different statistical methods, for example, Bland–Altman LoA [16, 17], ICC [18], Kappa coefficients [19], amongst others.

Some agreement studies may be constrained by the sample size available, for example when embedded within studies powered on a different outcome, or the pre-determined target sample may not be achieved for financial, temporal or other reasons. Nevertheless, the target and actual samples used should still be described and justified. The quality of agreement studies could be improved by following the Guidelines for Reporting Reliability and Agreement Studies (GRAAS) recommendations [1], which require explanation for the chosen sample size and explicit reporting of the number of raters, subjects/objects and replicate observations.

Strengths of this review are that this is the first to investigate how typical sample sizes in recent medical agreement studies differ by field, types of endpoints and statistical method. A team of statisticians was involved in the assessment of studies, allowing for increased accuracy of data review and extraction, and reduction of bias. Limitations include the use of only one electronic repository; research studies not present within the PubMed registry would not have been captured. Relatively few search terms were used, meaning some relevant studies may have been missed. Searches were limited to English language, meaning studies in other languages were also not included.

## Conclusions

We reviewed clinical agreement studies and noted that typical sample sizes varied according to research area, statistical approach and type of endpoint. We found that for continuous and categorical endpoints, the median sample sizes for agreement analyses were 50 (IQR 25 to 100) and 119 (IQR 50 to 271), respectively.

A sample size justification should be provided in all research studies even if a formal sample size calculation is not possible. However, despite the importance of a sample size justification, we found that only a third of papers reporting agreement studies provided one. The quality of reporting of agreement studies would be improved by following the guidelines in the GRAAS checklist [1] as this includes an item requiring an explanation as to how the sample size was chosen.

## Supplementary Information

**Additional file 1: Supplementary Table 1.** Distribution of sample sizes by field of study and type of endpoint. **Supplementary Table 2.** Distribution of sample sizes by statistical methods and type of endpoint.

**Additional file 2. **

## Data Availability

The dataset analysed during this study is included in Supplementary File 2.
