# Supplementary material for: A descriptive study of samples sizes used in agreement studies published in the PubMed repository
Source: BMC Med Res Methodol. 2022 Sep 19;22:242. doi: 10.1186/s12874-022-01723-5 (PMC9487062; doi:10.1186/s12874-022-01723-5)
Supplement: Supplementary file 1 — Additional file 1: Supplementary Table 1. Distribution of sample sizes by field of study and type of endpoint. Supplementary Table 2. Distribution of sample sizes by statistical methods and type of endpoint. [file 12874_2022_1723_MOESM1_ESM.docx]

Supplementary File 1

**Supplementary Table 1: Distribution of sample sizes by field of study and type of endpoint**

|  | |  | | Sample Size reported by studies | | | | |
| --- | --- | --- | --- | --- | --- | --- | --- | --- |
|  |  | n | | Median | | Mean | Interquartile  range | Range |
| Medicine | Categorical | | 13 | 150.0 | 308.6 | | [60.5, 271.0] | [30, 2000] |
|  | Continuous | | 24 | 54.5 | 77.9 | | [31.0, 100.5] | [11, 265] |
|  | Both | | 8 | 72.5 | 721.6 | | [59.0, 500.5] | [40, 4469] |
|  |  | |  |  |  | |  |  |
| Surgery | Categorical | | 1 | 3706.0 | 3706.0 | | NA | NA |
|  | Continuous | | 3 | 24.0 | 39.0 | | NA | [13, 80] |
|  | Both | | 0 | NA | NA | | NA | NA |
|  |  | |  |  |  | |  |  |
| Radiology | Categorical | | 13 | 57.0 | 367.2 | | [43.0, 273.0] | [10, 3082] |
|  | Continuous | | 16 | 41.0 | 75.4 | | [24.0, 103.0] | [14, 259] |
|  | Both | | 0 | NA | NA | | NA | NA |
|  |  | |  |  |  | |  |  |
| Allied Health | Categorical | | 1 | 56.0 | 56.0 | | NA | NA |
|  | Continuous | | 3 | 50.0 | 83.0 | | NA | [40, 159] |
|  | Both | | 0 | NA | NA | | NA | NA |

**Supplementary Table 2: Distribution of sample sizes by statistical methods and type of endpoint**

|  | |  | Sample size reported by studies | | | |
| --- | --- | --- | --- | --- | --- | --- |
|  |  | n | Median | Mean | Interquartile range | Range |
| Bland-Altman Limits of Agreement (n=41) | Categorical | 0 | NA | NA | NA | NA |
|  | Continuous | 36 | 65.5 | 87.0 | [32.0, 124.0] | [12.0, 265.0] |
|  | Both | 5 | 65.0 | 103.4 | [47.0, 179.0] | [40, 278] |
|  |  |  |  |  |  |  |
| Intra-class Correlation Coefficient (n=29) | Categorical | 1 | 36.0 | 36.0 | NA | NA |
|  | Continuous | 22 | 35.0 | 42.6 | [24.0, 50.0] | [12, 106] |
|  | Both | 6 | 72.5 | 909.2 | [64.0, 723.0] | [54, 4469] |
|  |  |  |  |  |  |  |
| Kappa Coefficient (n=35) | Categorical | 23 | 107.0 | 499.5 | [50.0, 275.0] | [10, 3706] |
|  | Continuous | 5 | 70.0 | 79.6 | [32.5, 131.5] | [16, 164] |
|  | Both | 7 | 65.0 | 186.3 | [54.0, 278.0] | [40, 723] |
|  |  |  |  |  |  |  |
| Significance Test (n=20) | Categorical | 5 | 150.0 | 140.4 | [44.5, 231.5] | [39, 267] |
|  | Continuous | 14 | 54.5 | 70.1 | [20.0, 99.0] | [12, 259] |
|  | Both | 1 | 40.0 | 40.0 | NA | NA |
|  |  |  |  |  |  |  |
| Others (n=32) | Categorical | 11 | 129.0 | 736.4 | [56.0, 465.0] | [10, 3706] |
|  | Continuous | 18 | 48.0 | 48.3 | [29.0, 61.0] | [11, 102] |
|  | Both | 3 | 278.0 | 1604.0 | NA | [65, 4469] |
